# Bioactive Characterization of Packaging Foils Coated by Chitosan and Polyphenol Colloidal Formulations

**DOI:** 10.3390/ijms21072610

**Published:** 2020-04-09

**Authors:** Sanja Potrč, Meta Sterniša, Sonja Smole Možina, Maša Knez Hrnčič, Lidija Fras Zemljič

**Affiliations:** 1 Laboratory for Characterization and Processing of Polymers, Faculty of Mechanical Engineering, University of Maribor, Smetanova 17, SI-2000 Maribor, Slovenia; 2 Faculty of Chemistry and Chemical Engineering, University of Maribor, Smetanova 17, SI-2000 Maribor, Slovenia; masa.knez@um.si; 3 Department of Food Science and Technology, Biotechnical Faculty, University of Ljubljana, Jamnikarjeva101, SI-1000 Ljubljana, Slovenia; meta.sternisa@bf.uni-lj.si (M.S.); sonja.smole-mozina@bf.uni-lj.si (S.S.M.)

**Keywords:** food packaging, PE/PP functionalization, bioactive properties, antimicrobial, antioxidant, activity, chitosan, polyphenols

## Abstract

Polypropylene (PP) and polyethylene (PE) foils, previously activated by ultraviolet (UV)/ozone, were functionalized using chitosan-extract nanoparticle dispersions. A solution of macromolecular chitosan was applied onto foils as a first layer, followed by the deposition of various extracts encapsulated into chitosan nanoparticles, which were attached as an upper layer. Functionalized foils were analyzed from a bioactive point of view, i.e., regarding antimicrobial and antioxidant activity. Desorption kinetics were also studied. Moreover, barrier properties were examined, as the most important parameter influencing antimicrobial and antioxidant activity. Finally, all these properties were correlated with different surface parameters, determined previously, in order to understand if there is any direct correlation between surface elemental composition, surface charge, contact angle, or morphology and a specific bioactive property. It was shown that great bioactive properties were introduced due to the additive effect of antimicrobial chitosan and antioxidative plant extracts. Moreover, oxygen permeability decreased significantly, and the migration of polyphenols and chitosan from the foil surface was below the OML (overall migration limit), which is very important for food industry applications. Furthermore, surface properties of foils influence to some extent the desired bioactivity.

## 1. Introduction

Active packaging (AP) is an innovative approach to maintaining or prolonging the shelf-life of food products while ensuring their quality, safety, and integrity. Using active packaging, as opposed to traditional methods, proves advantageous in many respects. The use of modern solutions in the field of packaging can contribute to a significant improvement in the sensory characteristics of food products and may ensure their microbiological safety. Their application helps to prevent food spoilage and subsequent waste, and it enables longer transportation and storage times. Active packaging systems can be divided into active scavenging systems (absorbers) and active releasing systems (emitters). Whereas the former remove undesired compounds from the food or its environment, for example, moisture, carbon dioxide, oxygen, ethylene, or odor, the latter add compounds to the packaged food or into the headspace, such as antimicrobial compounds, carbon dioxide, antioxidants, flavors, ethylene, or ethanol. The addition of active substances such as antimicrobials and antioxidants with AP instead of direct addition to the food may decrease the amount of such substances required [1]. Traditionally, active substances are added to the bulk of the food, whereas, for most fresh and processed food, food degradation or microbial growth occurs at the surface of the food. Furthermore, the activity of the active substances when added directly to food may be reduced or inhibited, because of the interaction between the active substances and the food components, as well as interactions during the processing of the food. Therefore, the addition of active substances via active packaging may be more effective than their addition to the bulk of the food [2].

There were numerous studies carried out in recent years on antimicrobial packaging intended for food. Most of these solutions proposed numerous active substances, using diverse technologies for their incorporation into packaging materials to control the growth of microorganisms, effectively extending the shelf-life of food products. However, the commercially available antimicrobial packaging systems are mostly based primarily on substances containing silver, which was recently recognized as a toxic agent in specific forms and concentrations [3]. Other coatings of interest are substances with silver and zeolite that also work in an effective way (Microban^®^, AgIon^®^, and Irgaguard^®^). Traditionally, synthetic antioxidants, such as butylated hydroxyanisole (BHA), butylated hydroxytoluene (BHT), tertiary butyl hydroquinone (TBHQ), propyl gallate (PG), and organophosphate and thioester compounds, were used extensively to enhance the shelf-life of food products by providing oxidative stability. Torres-Arreola et al. reported that incorporation of BHT into low-density polyethylene delayed lipid oxidation and protein denaturation [4]. However, the presence of synthetic antioxidants in food can have potential toxic and carcinogenic effects, and strict statutory controls are required [5]. Currently, consumers are responding increasingly to the perceived health risks associated with synthetic substances with unhealthy side effects (such as silver, benzoic acids, and other synthetic antioxidants) in food and pharmaceutical products, and they are becoming more and more concerned and watchful for their health. The primary concern of all food processors should therefore be the safety of the food they produce and package in order to meet the needs of their customers. 

New types of plastics and antimicrobial substances were, thus, researched and developed. To avoid potential risks and meet the consumer need for safe natural foods, natural antioxidants such as plant extracts, tocopherol, or essential oils from herbs and spices can be used as alternatives to synthetic antioxidants, and they will likely be utilized widely in food packaging to confer antioxidant activity [6,7,8,9,10,11]. Several authors reported that better results could be obtained by incorporating the volatile components of essential oils into films or edible coatings, and enhanced results can also be achieved through the encapsulation of essential oils in polymers with edible and biodegradable coatings, sachets, or nano emulsions [12,13,14,15]. Although many essential oils show promising results because of their effects against microorganisms, some limitations were also identified in the application of essential oils in this kind of antimicrobial AP [16]. For example, Hyldgaard et al. [17] reported that the antimicrobial potency of essential oil constituents depends on the pH, temperature, and level of microbial contamination present in the food. In practice, much higher concentrations of essential oils were required (as a determined minimal inhibitory concentration) as coatings to achieve final antimicrobial effects [18]. In addition, the use of essential oils in higher concentrations may cause negative sensory effects because of their intense aroma, which partially limits their use as preservatives in food packaging. To alleviate these problems, smaller amounts of essential oils can be combined with other antimicrobial compounds. One of the intelligent approaches could be the combination of extracts with polysaccharides as a coating for films [19,20,21,22,23,24] in order to positively change their mechanical, optical or barrier properties and to introduce bioactive properties in a suitable way. The use of antioxidants in synergistic formulation with other biopolymers as nanoparticles, with enlarged contact area compared to conventional antioxidant agents, might allow for a reduction in the amount of active substances, which would act very efficiently while simultaneously reducing and/or avoiding the effect of active substances on the required properties of the base packaging material [25,26,27]. Chitosan, obtained from chitin by partial deacetylation, which makes it soluble in acidic aqueous solutions, has diverse, industrially important properties, such as biocompatibility, biodegradability, and non-toxicity. Among many attractive features of chitosan, its safety profile compared with other natural polysaccharides is one of the most important, especially in food packaging applications, where it is necessary to monitor the concentration of toxins and endotoxin to ensure food safety. The purity of chitosan is essential for its toxicological profile. However, the United States (US) Food and Drug Administration (FDA) approves the use of chitosan as a food additive, with the status GRAS (generally recognized as safe). Therefore, chitosan has an advantage compared to other synthetic polymers, which is reflected in its widespread uses as edible film and food packaging material [20].

However, all of these concepts call for a detailed study in order to understand coating behavior in such a way as to assure a balance between bioactive efficiency and other required physico-chemical properties of base materials, such as mechanical properties, barrier properties, hydrophilicity, etc. One of the most important ones is to assure that the migration profile is in accordance with the required overall migration limit (OML) of a compound from surfaces, which has to be below 10 mg/dm^2^ or 60 mg/kg food in line with the Guidance Documents on (European Union (EU)) 10/2011 [28]. All these phenomena creating a bioactive character may be strongly dependent on the surface properties of foils that create the interface point in the contact with food, and they are mainly responsible for the final bioactivity of AP material.

In our previous work, the first part of this complex study, we reported the smart approach for the development of active packaging for two representative, commercially used foils, polypropylene (PP) and polyethylene (PE), previously activated by ultraviolet (UV)/ozone, using chitosan-extract colloidal dispersions as a coating for foils [23]. A solution of macromolecular chitosan was applied as a first layer onto PE and PP foils, followed by the deposition of various extracts (rosemary, thyme, and cinnamon) encapsulated into chitosan nanoparticles as an upper, second layer. Higher stability and homogeneity of the coating were obtained in this way. It was shown [23] that analyzed physico-chemical parameters such as surface charge, elemental composition, hydrophilicity/hydrophobicity, and morphology were promising for providing good bioactive properties. The latter hypothesis was verified in this paper. Functionalized foils were therefore analyzed with respect to antimicrobial effectiveness and antioxidative activity as well as desorption kinetics. Moreover, barrier properties were examined as well, as the most important parameter influencing antimicrobial and antioxidant activity. Finally, all these properties were correlated with different surface parameters to understand whether there is a direct correlation or dependence between the composition of the surface elements, surface charge, contact angle or morphology and a specific bioactive property.

## 2. Results and Discussion

### 2.1. Dispersion Characterization

#### 2.1.1. Minimal Inhibitory Concentration (MIC)

Determined MICs of thyme, rosemary, and cinnamon extracts are shown in Table 1. All three extracts showed antimicrobial activity, with lower values obtained for both bacteria, and with the best antimicrobial activity seen in rosemary extract, as noted in previous studies [29]. Likewise, thyme and cinnamon are known to be effective antibacterial [30], as can also be seen from our results. Antifungal activity was less pronounced, while, for rosemary and cinnamon extract, no MICs could be determined, due to the low solubility of these extracts and the impossibility of preparing a higher concentration of extract solution without the growth of fungi being affected by the solvent. Antifungal activity is generally less pronounced than antibacterial, and, from extensive research of natural antifungals, thyme, rosemary, and cinnamon are among the most active sources [29,31]. The concentration of the extract in the coating solution was determined according to the MICs. The final amount of the extract, which was encapsulated into chitosan nanoparticles and further applied onto the foil surface, was 4× its MIC, in order to obtain good antimicrobial activity of functionalized foils.

#### 2.1.2. Total Phenolic Content

The total phenolic contents of thyme, rosemary, and cinnamon, as well as of chitosan nanoparticles with embedded extracts, were already reported and discussed in our previous paper [23]. It was shown that the concentration of phenols in extract solutions was the highest in cinnamon, followed by rosemary and then thyme. The total phenolic content was significantly lower in chitosan nanoparticle (CSNP) formulations, which again proves that the extracts were encapsulated in the interior of chitosan nanoparticles, and, due to their poor solubility, they retained the embedded extracts. Furthermore, chitosan itself shows very poor antioxidant behavior and, thus, it has no significant effect on the phenolic content. The highest total phenolic content was observed for chitosan nanoparticles with embedded cinnamon extract (CSNPs CIN), which correlates with the result for pure cinnamon, as the richest in phenolic substances [23].

### 2.2. Functional Foils

#### 2.2.1. Oxygen Permeability

Table 2 reports oxygen transmission rates for untreated reference PE and PP foil and functionalized foils, which is an extremely important property to control food spoilage. It can be seen that the oxygen permeability was reduced in all cases after coating procedures. In almost all cases, the reduction was higher than 50%. In general, raw PP foils have lower oxygen transmission rate (OTR) than PE foils; however, on the other hand, oxygen permeability decreased more after functionalization of PE foils compared to PP foils. For example, in the case of coating with cinnamon extract embedded into chitosan nanoparticles onto the first chitosan macromolecular solution layer, PE foil exhibited 61% reduction of oxygen permeability, and a 46% decrease was observed for PP foil. This may be due to the more homogeneous and thin-film coating on PE foil, as revealed by the SEM results [23]. It was shown that PE exhibited a more hydrophilic nature than PP after UV/ozone pretreatment [23]. Therefore, better adhesion of polar CS and CSNP solutions was obtained for the PE foil and, consequently, a more homogeneous coating, leading to a more extensive reduction of oxygen permeability. The highest decrease in oxygen permeability (76% ± 3%) belonged to the PE foil coated with 2% chitosan solution and chitosan nanoparticles with incorporated thyme extract. It was shown recently, for both PP and PE foils coated with 2%CS + CSNPs THY (thyme), that rough coating appeared on foils with visible particles on the surface, which formed cross-linked or branched agglomerates and covered the foil surface uniformly. SEM results reported in Reference [23] showed that foils coated with 2%CS + CSNPs CIN expressed the most homogeneous and smoothest adsorbates, as well as covering the foil uniformly with less observed agglomerates. Thus, from a structural point of view, one would expect here the most expressive barrier properties for oxygen. However, it should be noted that the thickness and homogeneity of the first adsorbed layer, i.e. the macromolecular chitosan solution, also has a strong influence on the final barrier properties. 

**Figure 1:**
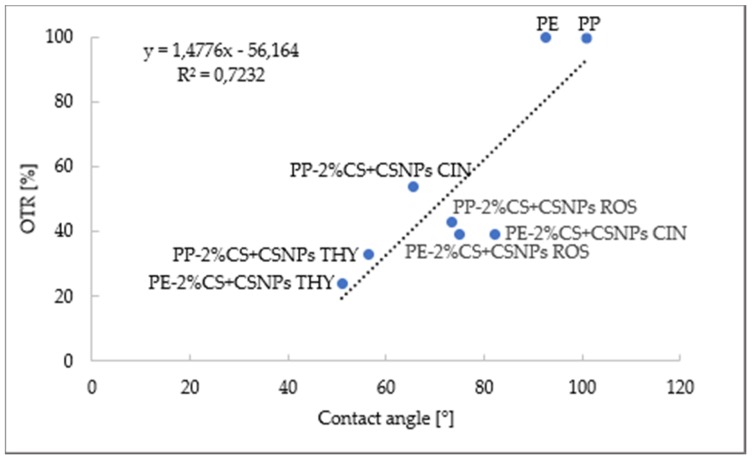
The correlation between contact angle and OTR.

The correlation between OTR (%) and contact angle, previously analyzed in Reference [23], is given in Figure 1.

Figure 1 shows that there is a good correlation between the OTR and the contact angle. Generally, a value of coefficient greater than 0.7 is considered as a strong correlation. It may be concluded that the hydrophilic character (contact angle below 90°) of the foil surface resulted in a better reduction of OTR (%) as expressed by their direct dependency.

#### 2.2.2. Desorption Experiment

Three methods, namely, polyelectrolyte titration, UV–visible light (UV–Vis) spectroscopy, and X-ray photoelectron spectroscopy (XPS), were used to evaluate the migration of functional coatings from the surfaces of polyethylene and polypropylene foils. The latter is very important to be studied, since the migration profile influences the bioactivity, as well as the sensorial properties of food.

##### Polyelectrolyte Titration

**Figure 2:**
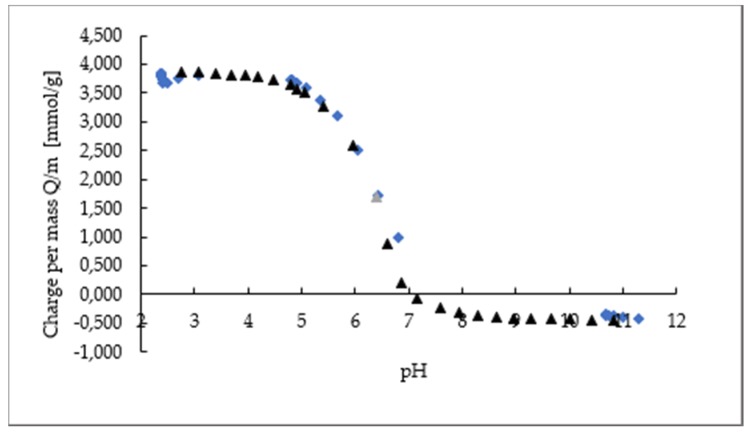
Potentiometric titration curves of chitosan: black curve, forward (from acidic to alkaline); blue curve, backward (from alkaline to acidic).

The amount of protonated amino groups of chitosan origin in different desorption baths was determined by polyelectrolyte titration. The results of chitosan desorbed from the surface of PE and PP are presented in Table 3. For this purpose, strictly, samples coated only with chitosan (2% CS as a first layer) were selected, whilst the presence of extract may influence polyelectrolyte titration mistakes and wrong determination of equivalence points. As can be seen, the migration of chitosan at pH 5.8 after 24 h (simulated pH of many of the food products such as toast, meat, etc.) was very low. This can be related to the fact that, at this pH value, not all of the amino groups were protonated; consequently, chitosan was insoluble. It may be seen from the potentiometric titration curves of chitosan macromolecular solution (Figure 2) that, at pH 5.8, only 50% of amino groups are protonated. The same behavior may be expected for chitosan nanoparticles onto foils. This may be explained with our previous work results, where it was seen clearly through measurement of zeta potential that, at this pH, for both foils, i.e., PE and PP coated only with a 2% chitosan macromolecular solution and these foils further coated with chitosan nanoparticles alone, the same charging behavior occurred [23]. Additionally, it may be seen from the zeta potential measurements that, at pH = 5.8, a level of 60% protonated amino groups in regard to a positive equilibrium plateau level was obtained for PE samples (PE-2% CS + CSNPs), while a level of 40% was obtained for PP-2% CS + CSNPs samples.

The low migration of chitosan can also be associated with the pre-activation of PE and PP foil by UV/ozone, which improves the interactions between this polysaccharide and PE or PP. In our previous work it was found that new oxygen-based groups (aldehyde and carboxyl groups) were introduced to the film surfaces, which can serve as binding sites for amino groups of chitosan (Coulomb interactions). 

**Figure 3:**
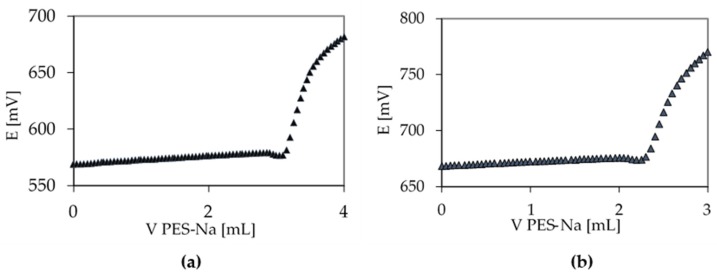
Polyelectrolyte titration curves of: PE-2%CS + CSNPs (**a**) and PP-2%CS + CSNPs (**b**).

The comparison between PE and PP foil coated with macromolecular chitosan solution and, further, by pure chitosan nanoparticles (as a reference in the case of extract encapsulation) showed a 37% higher migration of chitosan from the PE surface. For PE, 5.2 µmol of amino group per gram of foil was desorbed, and, for PP, 3.8 µmol of amino group per gram of foil was desorbed. The results are in the range of three replicates. As shown in our latest research [23], after the UV/ozone activation process, the PE foil showed a more hydrophilic character than PP, which indicates better adhesion of chitosan solutions onto PE. The latter statement was also proven by zeta potential measurements, which indicated a full coverage of PE by the chitosan macromolecules, whilst, for PP foils functionalized by chitosan, an incomplete surface coverage was suggested as seen from its lower isoelectric point (IEP). A higher amount of adsorbed chitosan may also be the reason for a slightly higher desorption (PE sample). However, it should be noted that all values are below the overall migration limit (OML) [28], which is extremely important for real use. The examples of titrations are shown in Figure 3.

##### Desorption of Extracts Using UV–Vis Spectroscopy

Thyme, rosemary, and cinnamon extract migration from the foil surface was evaluated using UV–Vis spectroscopy. The content of the extract in the desorption bath (pH 5.8) indicates how much extract was desorbed per kilogram of PE and PP foil after 24 h. If PE foils (Table 4) are compared with PP foils (Table 5), it can be seen that fewer polyphenols (extracts) were desorbed from the polyethylene foil. This confirms better adhesion of the extracts onto the PE. The latter may also be combined with the zeta potential results pointed out in Reference [23], which showed lower availability of polyphenols on the PP surface; consequently, a lower amount may be desorbed.

For all functionalized foils, it was found that desorption of extract was below the OML of 10 mg/dm^2^. Specifically, 0.023 mg/dm^2^ and 0.024 mg/dm^2^ of the thyme extract, 0.009 mg/dm^2^ and 0.015 mg/dm^2^ of the rosemary extract, and 0.003 mg/dm^2^ and 0.005 mg/dm^2^ of the cinnamon extract was desorbed from PE and PP, respectively. The highest migration was obtained for thyme and, contrarily, the highest amount of cinnamon was retained on the foils. The latter is in accordance with the isoelectric point shift, as an indication of successful binding of those foils’ curves (zeta potential as a function of pH) to more acidic regions. The highest shift in comparison to the foils coated with chitosan only was obtained for thyme–chitosan-coated foils, and the lowest was obtained for cinnamon–chitosan-coated foils [23].

In our opinion, the desorption would be much higher if the foils were not functionalized in the following way: (i) pretreatment of the foil with UV/ozone, and (ii) application of 2% chitosan solution as a first layer. The macromolecular solution of chitosan allows better adhesion of chitosan nanoparticles with the embedded extract and, thus, improved stability of the polyphenols on the foil surface. Moreover, in our approach, coatings exist from nanoparticles dispersed in the remaining chitosan and extract solution, with the strategy that an increase in stability can be achieved by simply incorporating the nanoparticles into another macromolecular layer [20].

##### Overall Desorption from PE and PP by XPS

Surface chemical compositions of two-layer coated PE/PP foils before and after the desorption experiment (24 h in Milli-Q water, pH = 5.8) were also investigated by XPS. This method was used to determine overall migration directly from foils, i.e., desorption of chitosan and polyphenols. Before XPS measurements, foils were dried at room temperature. The results collected in Table 6, Table 7 and Table 8 confirmed the presence of chitosan and polyphenols on the foil surface after the desorption test, which was also expected, considering the results obtained by polyelectrolyte titration and UV–Vis spectroscopy.

**Figure 4:**
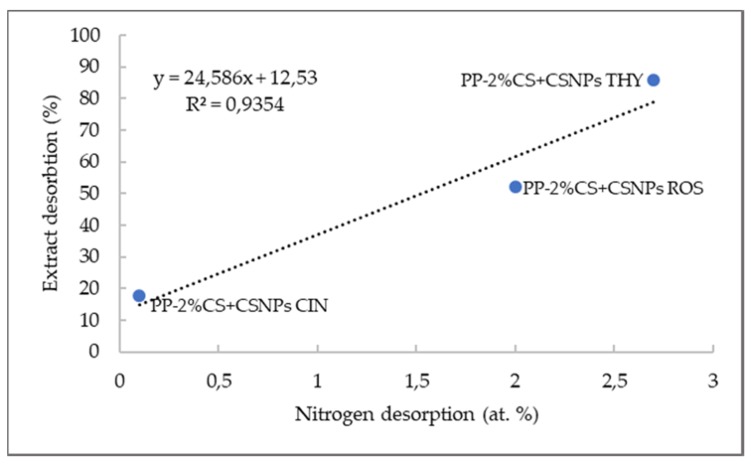
Correlation between nitrogen desorption and extract release.

The only source of nitrogen on the functionalized PE and PP surfaces was chitosan; thus, the stability of chitosan coating can be estimated from the atomic content of the nitrogen. It can be seen that the lowest concentration of chitosan was released from PP foil functionalized by chitosan macromolecular solution and CSNPs with embedded cinnamon extract; specifically, 0.1 at.% of N was released, which means that only around 3% of chitosan desorbed from the surface. The highest decrease in N was determined for PP foil functionalized by CSNPs with encapsulated thyme extract (i.e., from 4.8 at.% to 2.1 at.%), which may be due to partial coverage of the foil with the thyme nanoparticles (second layer) as revealed by SEM images [23]. This result is in accordance with indirect UV–Vis spectroscopy, which showed the higher migration of thyme extract from PP foil surfaces. The latter suggests clearly that the solubility of chitosan is the driving force for the extract release, discussed on the basis of Figure 4, which presents the correlation of desorbed N and released extracts for PP foils.

A very strong correlation between the extract release (in %) and nitrogen desorption (at.%) is seen from Figure 4. The correlation coefficient between both obtained values is 0.9354, which indicates that the amount of desorbed extract is directly proportional to the nitrogen desorption. This proves the fact that extracts are embedded into chitosan nanoparticles, and that the driving force for extract release is chitosan solubility. When chitosan is soluble, it may desorb from the foil surface, thus enabling extract release.

Moreover, the thyme nanoparticles do not cover the chitosan film completely, whilst cinnamon shows complete coverage of foil with nanoparticles. This means that, in the case of thyme, more amino groups of chitosan were exposed to the desorption process, which led to lower stability and, consequently, higher migration of the chitosan into the desorption bath. As can be observed, the film of nanoparticles acts as a good barrier for chitosan macromolecular desorption. When the nanoparticle dispersion was applied as a second layer onto the chitosan coating, chemical interactions were introduced and, therefore, the stability was improved.

Whereas chitosan was the only source of nitrogen, both organic substances, i.e., chitosan and polyphenol, are rich in oxygen. After the desorption experiment, the oxygen concentration decreased by 3–4 at.% (about 10%) for the thyme samples. Moreover, no significant changes in the oxygen concentration were detected for foils with cinnamon and rosemary application. This once again confirms the good stability of the coating on the foil surface. In order to achieve good stability of polyphenols, it is very important to ensure strong interactions between the chitosan and nanoparticles and a high encapsulation degree, which makes polyphenols more difficult to access.

Sodium and phosphorus are present in the chitosan nanoparticle structure, since they were synthesized using the crosslinker sodium tripolyphosphate. After desorption, the Na and P were not present on the foil surfaces, indicating the release of chitosan nanoparticles.

The results obtained by UV–Vis spectroscopy and polyelectrolyte titration showed a very good correlation with the XPS data. In the case of UV–Vis spectroscopy and polyelectrolyte titration, the analyzed samples were solutions from desorption baths and indirect determination was done, whereas XPS was used for direct determination, i.e., the concentration decrease in foil-specific elements. All three methods confirmed that the migration of polyphenols and chitosan from the foil surfaces was below the OML, which is very important for food industry applications.

#### 2.2.3. Bioactivity

##### Antimicrobial Activity

The antimicrobial properties of the functional coatings on PE and PP foils against Gram-positive bacteria *Staphylococcus aureus* and Gram-negative bacteria *Escherichia coli* are presented in Table 9, and those against fungi *Aspergillus flavus* and *Penicillium verrucosum* are summarized in Table 10.

Complete bacterial inhibition was shown in the case of PE and PP foils, coated with 2% chitosan solution and chitosan nanoparticles with incorporated rosemary extract. Furthermore, for foils functionalized by nanoparticles with embedded cinnamon and thyme extract, over one logarithmic value reduction in bacteria count was achieved; PE and PP reference foils showed no effect on bacteria. For inhibition of fungi, cinnamon and rosemary extract as coatings embedded into chitosan nanoparticles showed comparable inhibition with higher antifungal activity against *A. flavus* (3–4 logarithmic values) as compared to *P. verrucosum* (2–3 logarithmic values). Thyme was shown as the least active against both fungi, but with better activity against *P. verrucosum*. Overall, foils functionalized by nanoparticles with embedded rosemary extract proved to be the most effective against bacteria and fungi. High antimicrobial activity can be attributed to the synergistic effect of chitosan and polyphenol extracts [32], which can be even more important in fungi, where the effect of the extracts themselves was not as evident as in foil testing. Plant extracts are rich in different polyphenolic compounds [33], which have a synergistic action between them, while it is also possible with chitosan amino groups [32]. These compounds have several targets for action against microorganisms, like increased membrane permeability, inhibition of efflux pumps, damage to cytoplasmic membranes, interference with the cellular generation system, disruption of the proton motive force, interference with gene expression, and inhibiting RNA synthesis [34,35].

**Figure 5:**
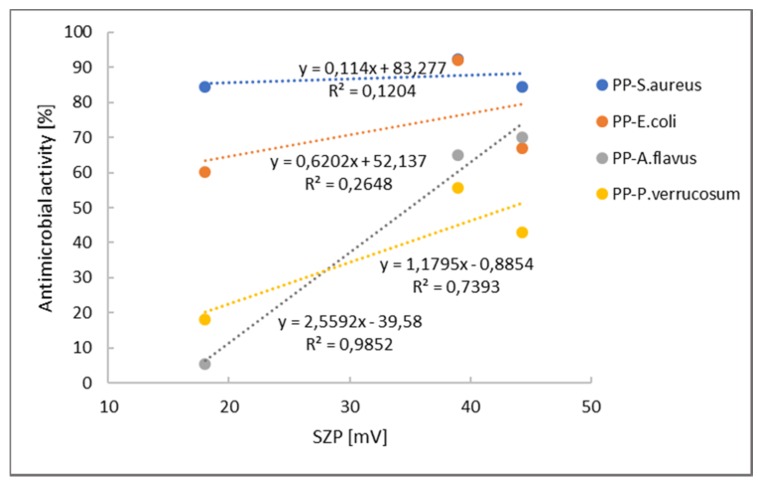
Correlation between positive zeta potential plateau level and antimicrobial activity.

From the results of the minimum inhibitory concentration, it can be seen that the extracts show an antimicrobial effect even at low concentrations, especially rosemary, which also has a high content of total phenols [23]. In the case of chitosan, protonated amino groups are those which, primarily, induce an antimicrobial effect. Moreover, it was shown previously that higher amounts of available protonated amino groups indicate an increased antimicrobial efficacy [36]. Thus, correlation was done between the positive zeta potential plateau level as an indicator of amino group quantity and a reduction in R (antimicrobial activity) (Figure 5).

Figure 5 indicates that there is no correlation between the antimicrobial activity and positive ZP plateau level for bacteria. A coefficient lower than 0.4 is considered as weak or no correlation. In both cases, for *S. aureus* and *E. coli,* the coefficients are below 0.2 and 0.3, respectively. Oppositely, quite good correlation may be seen for fungi, *A. flavus* and *P. verrucosum*. It seems that for inhibition of fungi, the content of amino groups is important, whilst some other mechanisms are also involved for bacteria inhibition. The most accepted mechanism for microbial inhibition is the electrostatic interaction between positively charged chitosan molecules and negatively charged microorganism surfaces. Consistent with such a mechanism, antimicrobial activity will be greater if more cationic sites are available [37]. Additionally, another proposed mechanism is the binding of chitosan with microbial DNA, which leads to the inhibition of the messenger RNA (mRNA) and protein synthesis via the penetration of chitosan into the nuclei of the microorganisms. The third possible mechanism is the chelation of metals, suppression of spore elements, and binding to essential nutrients for microbial growth. It is well known that chitosan has excellent metal-binding capacities, where the amino groups in the chitosan molecules are responsible for the uptake of metal cations by chelation [38]. In general, such a mechanism is more efficient at high pH, where chitosan’s amino groups are unprotonated, and the electron pair on the nitrogen atom is available for donation to metal ions [37].

For most polyphenols, it was shown that the antibacterial activity likely depends on interactions between polyphenols and the bacterial cell surface [39], where the main descriptors included in antimicrobial activity are the lipophilicity and the electronic and charge properties of the polyphenols.

**Figure 6:**
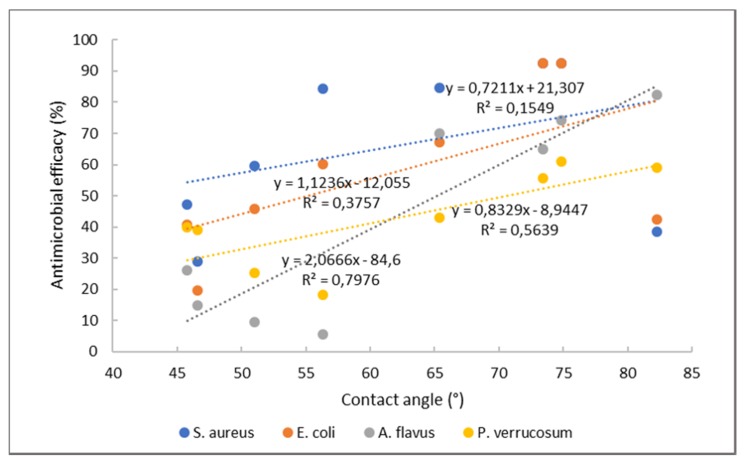
Correlation between contact angle and antimicrobial efficacy.

To gain even more insight into this, the antimicrobial activity was correlated with the contact angle (Figure 6). In general, it may be seen that, with the increase in contact angle, the antimicrobial activity increases as well. It was already shown that hydrophobic and super-hydrophobic surfaces are very effective for microbial reduction [40]. However, better correlation between both parameters may again be seen for fungi, whilst, for bacteria, again, poor or no correlation is suggested. Faster antimicrobial action of chitosan against fungi as compared to bacteria was reported previously [41]. One can speculate that surface properties of foils do not irritate the bacteria, whilst they alter fungal growth with suppression of sporulation and spore germination [42]. Obviously, as already suggested by bacteria inhibition with chitosan, the chelation mechanism and an interaction with DNA are involved, whilst, for fungi, the inhibition may occur through an electrostatic interaction of the foil surface and fungal cell wall.

Comparing PE and PP with the same coating, it can be seen clearly that coatings on PP foil are more effective against bacteria than the same coatings on PE foil. This may be due to better adhesion and a more homogeneous application on PP foil. In the case of fungi, there are no visible differences when observing the effectiveness of coatings on PE or PP foil. As expected, bacterial testing showed that it is preferable to have a dispersion of chitosan nanoparticles with encapsulated extract as the second layer, also due to the effect of the free protonated amino group. On the contrary, a better antifungal effect is achieved if polyphenols from the extract are available on the foil surface and, thus, this formulation is the driving force for fungal inhibition.

Good antimicrobial properties indicated that different combinations of chitosan and extracts might be applied on PE and PP foil as active packaging materials to prolong the shelf-life of different types of food.

##### Antioxidative Activity by ABTS Assay

**Figure 7:**
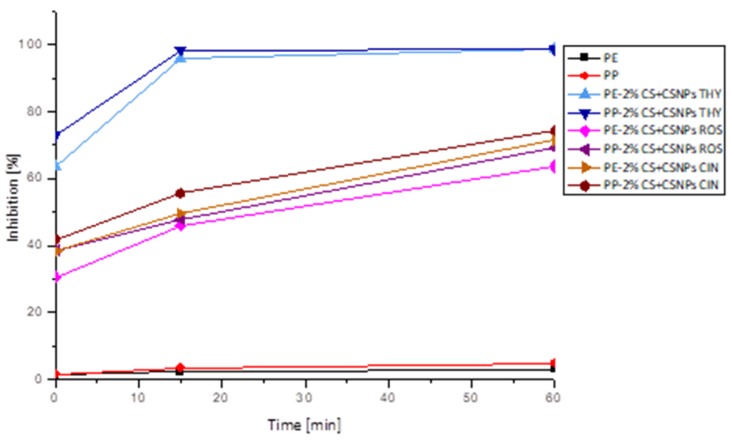
Antioxidative activity of reference PE and PP and ultraviolet (UV)/ozone-activated PE and PP foils, coated with 2% chitosan and chitosan nanoparticles with incorporated thyme/rosemary/cinnamon extract.

The antioxidant activity of functionalized PE and PP foils was evaluated using the ABTS radical cation-scavenging assay. PE and PP foils showed very poor antioxidant properties (3.0% and 4.5% inhibition). As shown in Figure 7, chitosan-based coatings which incorporated different polyphenols exhibited significant free-radical scavenging activity.

**Figure 8:**
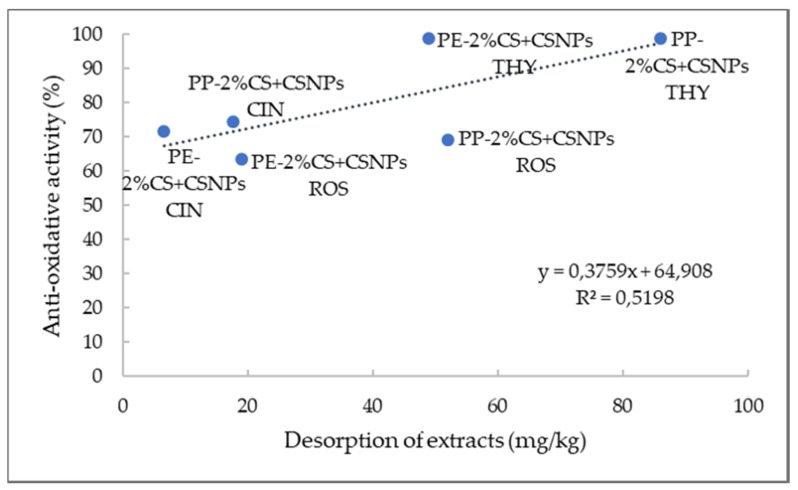
Correlation between amount of released extract and antioxidative activity.

**Figure 9:**
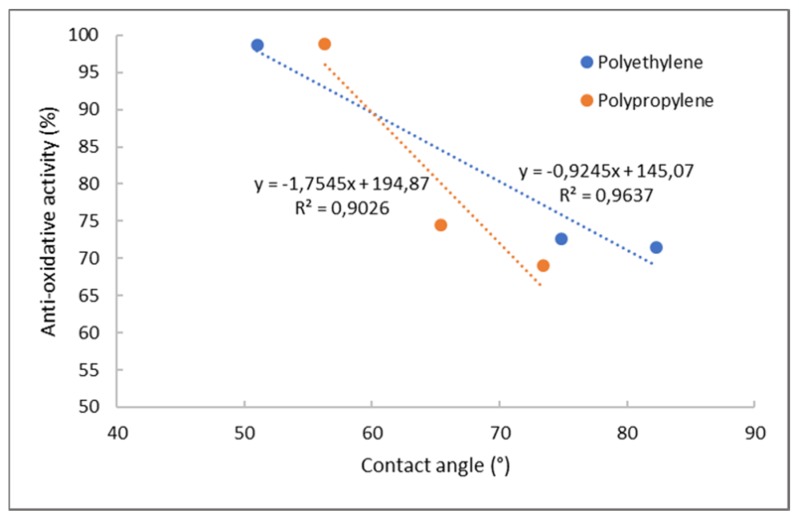
Correlation between contact angle and antioxidative activity for PE and PP foils.

Complete inhibition was achieved with the application of thyme extract. Already after 15 min, both foils showed the maximum free-radical reduction. Moreover, high antiradical activity was obtained for PE and PP foils coated with rosemary and cinnamon extract. In the case of cinnamon–chitosan nanoparticles applied on PE foil (PE-2%CS + CSNP CIN), 48.23% inhibition was determined after 15 min, and 63.26% inhibition was determined after 1 h. Antioxidant activity of the same coating attached onto PP foil was a little bit higher (faster kinetics) than for PE foils (55.21% after 15 min and 69.31% after 1 h). This may be due to better adhesion or a higher amount of available extract on the surface. Similar antioxidative inhibition can also be observed for the application of chitosan nanoparticles with embedded rosemary extract (PE/PP-2%CS + CSNP ROS). It should be noted that CS/sodium tripolyphosphate (TPP) extract dispersions were applied as a second layer, and, because the extracts attached not only inside the particles, but also on the particle surfaces, this led to higher antiradical activity. The latter is also very much connected to the released amount of extracts (Figure 8), where solid correlation between antioxidative activity and desorption of extracts was noted (correlation coefficient of 0.5198). When antioxidative activity is correlated to the contact angle (Figure 9), it may be seen that the higher contact angle worsens antioxidant activity. This is also in accordance with the results of correlation between OTR and contact angle, where the same trend was obtained, i.e., a higher contact angle increases OTR. From this, it may be concluded that antioxidant activity is also reduced with higher oxygen permeability. The latter is logical, as the presence of oxygen causes oxidation processes which worsen storage conditions. However, the contrary may be seen for antimicrobial activity where more complex mechanisms are involved.

Remarkable antioxidative properties can be mainly attributed to the high content of polyphenols in thyme, cinnamon, and rosemary extracts, which contain large amounts of phenolic hydroxyl groups, which can donate hydrogen to free radicals [43]. Furthermore, chitosan film exhibits minimal, but not efficient, antiradical scavenger capacity against the ABTS^+^ radical, and its activity increases with the degree of deacetylation due to more amino groups [44]. This was also shown with our results; no efficient antioxidative inhibition may be seen with chitosan coating only (5% of scavenger capacity).

According to the promising results of functional coatings presented in this paper, a decrease in oxidative food deterioration can be expected.

**Figure 10:**
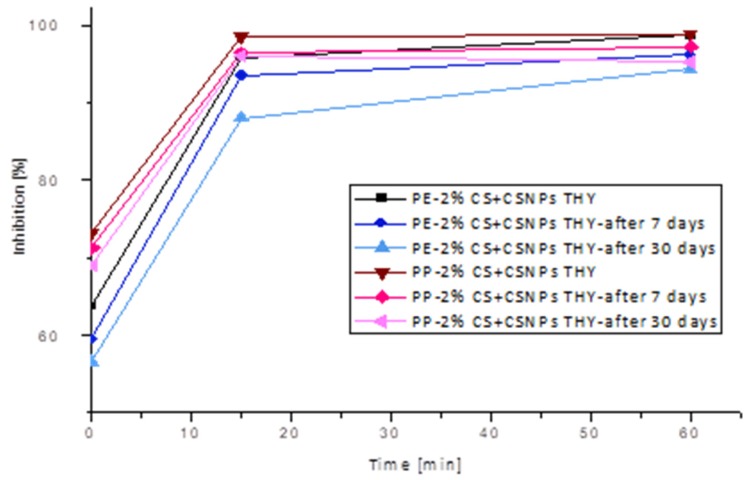
Antioxidative activity of UV/ozone-activated PE and PP foils, coated with 2% chitosan and chitosan nanoparticles with incorporated thyme extract directly after application, after seven days and after one month.

As can be seen, thyme extract has proven to be a very good antioxidant and the most promising coating embedded into chitosan nanoparticles. For this purpose, thyme extract was used for monitoring the antioxidant efficacy of functionalized foils one month after coating application. The results are presented in Figure 10, showing a very good stability of the CSNPs THY layer on polyethylene and polypropylene surfaces. After seven days, a 5% reduction of antioxidative activity for PE and a 3% reduction of antioxidative activity for PP were observed (compared to measurements directly after coating application); after one month, the reduction of inhibition was only 9% for PE and 7% for PP. Functionalized PP foils showed greater inhibition, which decreased slightly with longer storage. However, a larger difference between storage times occurred with functionalized PE foils, where radical scavenging reaction was slower with longer storage times. Nevertheless, results show clearly that antioxidant activity is very high even one month after coating application; functionalized PE and PP foils with thyme extract inhibited the radicals almost completely after 1 h (between 91% and 95%). For food safety and quality, it is very important to prevent its oxidation, and active packaging materials may prove to be a good solution.

## 3. Materials and Methods

### 3.1. Materials

Low-molecular-weight chitosan (50 to 190 kDa, LMW) and deacetylated chitin (75–85%) were obtained from Sigma-Aldrich (St. Louis, MO, USA). Sodium tripolyphosphate (TPP) (MW = 367.85 g/mol) was obtained from Acros Organics, Geel, Belgium. ABTS (2,2′-azino-bis(3-ethylbenzothiazoline-6-sulfonic acid) diammonium salt) was obtained from Sigma-Aldrich. Hydroalcoholic natural tincture of thyme (*Thymus vulgaris L.*), 10% *Thymus vulgaris L*., 90% ethanol (15%) was obtained from Soria Natural, Spain. Acetic acid (MW = 60.05 g/mol), ≥99.8% was obtained from Sigma-Aldrich. Ethanol (MW = 46.07 g/mol), 99.8% (GC) was obtained from Honeywell Sigma-Aldrich. A Milli-Q water: Milli-Q Direct Water Purification System with a 0.2 μm PES (polyethersulfone) High Flux Capsule Filter was used. Polyethylene (PE) normal quality, transparent, GSM (gram per square meter) = 46.28 g/m^2^ (thickness 50 µm, slipperiness 0.207) was obtained from Makoter d.o.o., Ljutomer, Slovenia. Polypropylene (PP) normal transparent oriented, GSM = 22.93 g/m^2^ (thickness 27 µm, slipperiness 0.278) was obtained from Manucor S.p.A., Sessa Aurunca, Italy.

### 3.2. Solution and Functionalized Foil Preparation

Chitosan solution (1 wt.%) was prepared by dissolving LMW chitosan powder in Milli-Q water. Glacial acetic acid was added dropwise until a pH of 4.0 was reached. Solutions were stirred for 24 h to enable chitosan dissolution. All extracts were dissolved in absolute ethanol. The concentration of each extract was determined according to minimal inhibitory concentration (MIC), and 4× the amount of determined MIC was used in order to achieve good antimicrobial properties. CSNPs with encapsulated thyme/rosemary/cinnamon extract were synthesized via an ionic gelation technique, using sodium tripolyphosphate (0.2 wt.% TPP) as a crosslinking agent. Then, 20.0 mL of extract solution and 40.0 mL of TPP solution were added dropwise to 40.0 mL of chitosan solution while stirring. The particles were formed spontaneously during continuous stirring for 1 h at room temperature. The chitosan solution was then kept in a refrigerator and it was stable up to three months. However, the formulations were always applied onto foils within a period of one week.

To achieve better adhesion of chitosan macromolecular solutions and colloidal dispersions onto the surface, PE and PP foils were activated by a Novascan UV/ozone surface cleaner. The foils were firstly cleaned ultrasonically in a bath of 70% (*v*/*v*) ethanolic solution for 5 min, and then dried in an oven at 40 °C. Then, they were exposed to UV/O_3_ surface treatment (PE for 15 min and PP for 20 min) at 25 °C. 

Foils were functionalized in two layers (layer-by-layer composition). The first layer was a 2% (*w*/*v*) chitosan macromolecular solution prepared as pointed out in our previous article, and the second layer was a dispersion of CSNPs with incorporated thyme, rosemary, or cinnamon extract; recipe details are given in Reference [23]. The coatings were applied using roll-to-roll printing by a Johannes Zimmer machine, Austria. After application of each layer, the foils were dried at room temperature. A chitosan macromolecular solution was applied as the first layer, due to its high antibacterial efficacy and for better adhesion of extract solutions, which exhibit high antioxidant and antifungal activity. A description of the samples is given in Table 11.

### 3.3. Methods

#### 3.3.1. Determination of Minimal Inhibitory Concentrations

Antimicrobial activity against *Staphylococcus aureus* ŽMJ72, *Escherichia coli* ŽM370, *Aspergillus flavus* ŽMJ25, and *Penicillium verrucosum* ŽMJ23 was evaluated in this study. All microorganisms were from the culture collection of the Laboratory for Food Microbiology at the Department of Food Science, Biotechnical Faculty Ljubljana, Slovenia (designation ŽM and ŽMJ).

The broth microdilution method following Klančnik et al. [45] was used to determine minimal inhibitory concentrations (MIC). Microtiter plates were used to prepare two-fold dilutions of extracts in broth (tryptic soy broth for bacteria, malt extract broth for fungi), to which standardized inoculum was added. The plates were then incubated at 37 °C (24 h) for bacteria, and at 25 °C (5–7 days) for fungi.

#### 3.3.2. Oxygen Permeability

The oxygen transmission rate (OTR) was measured using the oxygen transmission rate system PERME^®^ OX2/230, Labthink Instruments Co., Ltd. PR China, according to Standard GB/T 19789-2005 or ISO 15105-2:2003. The thickness of the raw materials PE and PP was given by producers. The same thickness was used in measurements. OTR values and coefficient values are average results obtained from testing of five measurements twice, changing positions of the measurement (chamber). Oxygen transmission rates were determined at 23 °C and 50% relative humidity (flux = 10 mL/min).

#### 3.3.3. Desorption Experiments

##### Preparation of Desorption Bath and Samples

Firstly, 0.5 g of PE and PP foils, coated with chitosan macromolecular solution and chitosan nanoparticles with embedded plant extract, were immersed for 24 h in 30 mL of Milli-Q water, and the pH was adjusted to 5.8 using 0.1 M hydrochloric acid. The solutions were then filtered, and the filtrates were used for polyelectrolyte titration and for UV–Vis spectroscopy measurements as indirect methods. The PE/PP-coated foils were, after the desorption experiment, dried at room temperature and investigated using XPS as a direct method.

##### Polyelectrolyte Titration

A Mettler Toledo DL 53 titrator with a 10-mL burette was used for evaluation of the chitosan desorption. Firstly, 50 µL of the polyelectrolyte titrant (polyethylene sulfonate sodium salt (PES-Na, c = 10 Mm) was added every 5–10 s. The analyte was composed of 30 mL of filtrated solution (desorbed chitosan), 9 mL of distilled water, and a few drops of indicator Toluidine blue O (Sigma-Aldrich). Before the titration, the pH was adjusted to 3.6 with HCl in order to establish full protonation of chitosan. The absorbance was measured as a potential change in mV with a Mettler-Toledo Phototrode DP660 at 660 nm. The concentration of NH_3_^+^ was determined from the equivalent volume (V) of the added polyethylene sulfonate sodium salt. Three replicates were performed for each sample.

##### UV–Vis Spectroscopy

The concentration of plant extracts in desorption bath after 24 h was determined using a UV–visible spectrophotometer (Agilent technologies, Cary 60 UV-vis). To calibrate the absorbance of the solution, the spectrum of water at pH 5.8 was employed as a reference. Absorbance was measured at 285 nm for thyme and rosemary and at 282 nm for cinnamon extract.

##### XPS

The surface composition of uncoated and coated polymer foils was compared through using a TFA XPS instrument (Physical Electronics Inc., Chanhassen, MN, USA). The ultimate pressure in the XPS vacuum chamber was ~6 × 10^−8^ Pa. The samples were irradiated with X-rays from monochromatic Al Kα1,2 radiation at 1486.6 eV. The diameter of the analysis area was 400 µm.

The photoelectrons were detected with a hemispherical analyzer located at a take-off angle of 45° with respect to the normal sample surface. Survey-scan spectra were acquired at a pass energy of 187.85 eV, while, for C 1*s*, individual high-resolution spectra were taken at a pass energy of 29.35 eV with a 0.125-eV energy step. An additional electron gun was used for charge neutralization. All spectra were referenced to the main C 1*s* peak of the carbon atoms, which was assigned a value of 284.8 eV. The spectra were analyzed using MultiPak v8.1c software (Ulvac-Phi Inc., Kanagawa, Japan, 2006) from Physical Electronics, which was supplied with the spectrometer.

#### 3.3.4. Evaluation of Antimicrobial Potential of Functionalized Foils

The antimicrobial potential of material was evaluated using a modified ISO22196 method for measuring antimicrobial activity on plastic surfaces (ISO, 2007). After one day of incubation at 37 °C for bacteria and 25 °C for fungi, the number of viable bacteria and spores was determined using the pour plate method, with plate count agar for bacteria and malt extract agar for fungi [20]. For each tested material, the number of viable bacteria and spores per cm^2^ of tested material was calculated, and antimicrobial activity (*R*) was calculated following Equation (1), where *U*t is the average of the logarithm of the number of cells/spores in the control, and *A*t is the average of the logarithm of number of cells/spores in treated foils. Where there was no visible bacterial growth on the plates, the volume of dilution was taken to calculate number of cells, and the final results were presented as “smaller than this value”.

*R* = *U*t − *A*t. (1)

#### 3.3.5. Antioxidative Activity (ABTS)

Antioxidative activity was determined using ABTS, which was dissolved in water to a 7 mM concentration. ABTS radical cation (ABTS ^· +^) was produced by reacting ABTS stock solution with 2.45 mM potassium persulfate (final concentration). The solution was stored in the dark at room temperature overnight. For the study of extracts and functionalized foil antioxidative activity, the ABTS^· +^ solution was diluted with phosphate buffer solution (pH = 7.4) to obtain an absorbance of 0.700 ± 0.020 at 734 nm. After that, 3.9 mL of diluted ABTS^· +^ solution was added to 0.1 mL of chitosan nanoparticles with encapsulated extract or to 0.1 g of functionalized foil. Absorbance was measured immediately, after 15 min, and after 1 h. Measurements for all samples and the reference ABTS radical cation solution were performed in triplicate. The percentage of radical scavenging activity was determined using Equation (2).

(2) $$Inhibition=\frac{Abs_{\mathrm{control}}-Abs_{\mathrm{sample}}}{Abs_{\mathrm{control}}}\times 100\%,$$

where *Abs*_control_ is the absorbance of ABTS∙^+^ solution in phosphate-buffered saline (PBS), and *Abs*_sample_ is the absorbance of sample (the remaining concentration of ABTS radical cation in the presence of extract/functionalized foil).

#### 3.3.6. Statistical Analysis

The statistical analysis of the data was performed using IBM SPSS 23 software. Analysis of variance (ANOVA) and post hoc Dunnett *t*-test were used, considering a significant difference between the samples and control when *p* < 0.05. Statistical analysis was performed in the test of antimicrobial activity. All data were expressed as means ± standard deviation.

## 4. Conclusions

It was shown that great bioactive properties may be introduced to foil surfaces due to the additive effect of antimicrobial chitosan, together with antioxidative plant extracts as an adsorbate for PP and PE. The method of UV/ozone activation of foils and additional functionalization by chitosan macromolecular solution as a first layer and chitosan nanoparticles with embedded extracts as a second layer shows great potential for different packaging applications, such as active packaging in the food industry. This may be attributed to the higher reduction of oxygen permeability of functionalized foils, as well as to microorganism reduction with simultaneous antioxidant activity. In general, for all functionalized foils, the oxygen permeability reduction was higher than 50% in comparison with reference foils. Comparing PE and PP with the same coating, it can be seen clearly that coatings on PP foil are more effective against bacteria than the same coatings on PE foil. This may be due to better adhesion and more homogeneous application on PP foil. In the case of fungi, there are no visible differences when observing the effectiveness of coatings on PE or PP foil. As expected, bacterial testing showed that it is preferable to have a dispersion of chitosan nanoparticles with encapsulated extract as the second layer, due to the effect of the free protonated amino group. On the contrary, a better antifungal effect is achieved if polyphenols from the extract are available on the foil surface and, thus, this formulation is the driving force for fungi inhibition. The most efficient antimicrobial efficient samples were both foils, PE and PP, coated by chitosan and further coated by chitosan nanoparticles with embedded rosemary. These samples also showed good antioxidant activity, whilst antioxidant superiority could be seen by the sample coated by chitosan and further coated by chitosan nanoparticles with embedded thyme. The results obtained by UV–Vis spectroscopy and polyelectrolyte titration showed a very good correlation with XPS data regarding the desorption of chitosan and extracts from foil surfaces. All three methods confirmed that the migration of polyphenols and chitosan from the foil surface was below the OML, which is very important for food industry applications.

It was also shown that surface properties of foils have some influence on the bioactive profile, which may clarify some working mechanism. It was shown that lower contact angle (below 90°) of the foil surface resulted in a better reduction of OTR (%). Moreover, the solubility of chitosan is the driving force for the extract release, i.e., a good correlation was obtained of desorbed N and released extracts for PP foils. The released amount of extracts also influenced antioxidative capacity, whilst a solid correlation was noticed between antioxidative activity and desorption of extracts (correlation coefficient of 0.5198). When antioxidative activity is correlated to the contact angle, it may be seen that a higher contact angle worsens antioxidant activity. 

Regarding antimicrobial activity, it was seen oppositely that, with the increase in contact angle, the antimicrobial activity increased as well, whilst the number of amino groups did not correlate with antimicrobial activity. The latter again supports that protonated amino groups are not the driving force for antimicrobial activity, but some other surface parameter, such as foil morphology, hydrophilicity/hydrophobicity, etc., creates the antimicrobial response with a combination of antimicrobial mechanisms that, through the synergistic effect of chitosan and extracts, improves antimicrobial activity.

In this research, the packaging materials consisted of bio-based polymers as foil coatings, made from raw materials originating from agricultural or marine sources, which include polysaccharides (chitosan) and natural polyphenols. From a sustainability perspective, it is crucial to reduce the environmental footprint of packaging materials, which means that a trade-off among the economic, environmental, and social aspects should be accepted. Active packaging solutions require the implementation of new technologies that must be competitive with existing ones and comply with regulations, which is reflected in additional costs and investments. In our case, this results in technical bottlenecks related to coating application. On the contrary, the environmental and social benefits in terms of reducing food waste and extending the shelf-life of food are gained. This indicates that compromises between costs from an economic perspective and benefits from an environmental point of view are necessary to achieve sustainable packaging solutions. The research work, thus, considers the aspect of using the biodegradable materials in an environmentally friendly manner by reducing packaging waste, facilitating the recycling of packaging material, and reducing food waste through the introduction of natural and biodegradable surface activators as active control for food quality and spoilage.

## Figures and Tables

**Table 1:** Minimum inhibitory concentrations (MICs) of thyme, rosemary and cinnamon extracts determined by microdilution method.

Microorganism	Thyme (mg/mL)	Rosemary (mg/mL)	Cinnamon (mg/mL)
***Staphylococcus aureus***	3.10	0.03	0.23
***Escherichia coli***	6.30	0.47	1.25
***Aspergillus flavus***	12.50	>6.25	>5.00
***Penicillium verrucosum***	6.30	>6.25	>5.00

**Table 2:** The oxygen permeability between the reference foils and foils with the application. OTR—oxygen transmission rate; PE—polyethylene; PP—polypropylene; CS—chitosan; CSNP—chitosan nanoparticle; THY—thyme; ROS—rosemary; CIN—cinnamon.

Sample	OTR (cm^3^/m^2^∙d)	OTR (%)	Permeability Reduction (%)
PE	3226 ± 62	100 ± 2	/
PP	1078 ± 36	100 ± 3	/
PE-2%CS + CSNPs THY	789 ± 35	24 ± 3	76 ± 3
PP-2%CS + CSNPs THY	359 ± 9	33 ± 1	67 ± 1
PE-2%CS + CSNPs ROS	1260 ± 8	39 ± 1	61 ± 1
PP-2%CS + CSNPs ROS	464 ± 10	43 ± 1	57 ± 1
PE-2%CS + CSNPs CIN	1250 ± 51	39 ± 2	61 ± 2
PP-2%CS + CSNPs CIN	580 ± 8	54 ± 1	46 ± 1

**Table 3:** Desorption of chitosan amino group per kilogram of foil.

Sample/Desorption of Chitosan	PE-2%CS + CSNPs	PP-2%CS + CSNPs
mmol/kg	5.2 ± 0.2	3.8 ± 0.1

**Table 4:** Desorption of extracts per kg of PE foil.

Extract	Thyme	Rosemary	Cinnamon
**Sample**	PE-2%CS + CSNPs THY	PE-2%CS + CSNPs ROS	PE-2%CS + CSNPs CIN
Desorption (mg/kg)	49.0 ± 1.0	19.0 ± 3.0	6.6 ± 0.1

**Table 5:** Desorption of extracts per kg of PP foil.

Extract	Thyme	Rosemary	Cinnamon
**Sample**	PP-2%CS + CSNPs THY	PP-2%CS + CSNPs ROS	PP + 2%CS + CSNPs CIN
Desorption (mg/kg)	86.0 ± 1.0	52.0 ± 4.0	17.6 ± 0.8

**Table 6:** Surface chemical composition of PE/PP-2%CS + CSNPs THY-coated foils before and after desorption experiment (at.%).

Sample	C	N	O	Na	P
PE-2%CS + CSNPs THY	62.2	4.1	31.5	0.3	0.6
PP-2%CS + CSNPs THY	59.3	4.8	33.4	0.1	1.1
After 24 h desorption: PE-2%CS + CSNPs THY	57.4	2.4	28.4	/	/
PP-2%CS + CSNPs THY	55.2	2.1	29.4	/	/
Difference: PE-2%CS + CSNPs THY	−4.8	−1.7	−3.1	−0.3	−0.6
PP-2%CS + CSNPs THY	−4.1	−2.7	−4.0	−0.1	−1.1

**Table 7:** Surface chemical composition of PE/PP-2%CS + CSNPs ROS-coated foils before and after desorption experiment in at.%.

Sample	C	N	O	Na	P
PE-2%CS + CSNPs ROS	66.7	4.0	26.8	0.4	0.2
PP-2%CS + CSNPs ROS	62.0	3.8	27.8	0.3	0.3
After 24 h desorption: PE-2%CS + CSNPs ROS	55.5	2.1	29.0	/	/
PP-2%CS + CSNPs ROS	53.0	1.8	29.3	/	/
Difference: PE-2%CS + CSNPs ROS	−11.2	−1.9	2.2	−0.4	−0.2
PP-2%CS + CSNPs ROS	−9.0	−2.0	1.5	−0.3	−0.3

**Table 8:** Surface chemical composition of PE/PP-2%CS + CSNPs CIN-coated foils before and after desorption experiment (at.%).

Sample	C	N	O	Na	P
PE-2%CS + CSNPs CIN	63.3	4.0	27.4	0.7	0.3
PP-2%CS + CSNPs CIN	62.2	3.2	28.4	0.2	0.1
After 24 h desorption: PE-2%CS + CSNPs CIN	62.5	3.3	27.9	/	/
PP-2%CS + CSNPs CIN	58.0	3.1	29.1	/	/
Difference: PE-2%CS + CSNPs CIN	−0.8	−0.7	0.5	−0.7	−0.3
PP-2%CS + CSNPs CIN	−4.2	−0.1	0.7	−0.2	−0.1

**Table 9:** Antimicrobial activity against Gram-positive (*Staphylococcus aureus*) and Gram-negative bacteria (*Escherichia coli*).

Material	Bacteria			
	*S. aureus*		*E. coli*	
	N	R	N	R
Control foil	5.33 ± 0.17	/	5.46 ± 0.16	/
PE-2%CS + CSNPs THY	2.19 ± 0.24 *	3.14	3.02 ± 0.45 *	2.44
PP-2%CS + CSNPs THY	0.85 ± 0.40 *	4.48	2.22 ± 0.79 *	3.24
PE-2%CS + THYME	3.86 ± 0.38 *	1.47	4.45 ± 0.24 *	1.01
PP-2%CS + THYME	2.86 ± 0.33 *	2.47	3.30 ± 0.16 *	2.16
PE-2%CS + CSNPs ROS	<0.40 *	>4.93	<0.40 *	>5.06
PP-2%CS + CSNPs ROS	<0.40 *	>4.93	<0.40 *	>5.06
PE-2%CS + CSNPs CIN	3.23 ± 0.18 *	2.10	3.24 ± 0.60 *	2.22
PP-2%CS + CSNPs CIN	0.81 ± 0.31 *	4.52	1.77 ± 0.27 *	3.69

N—number of bacteria/spores per cm^2^ of material; R—antimicrobial activity. * *p* < 0.001 for Dunnett *t*-test.

**Table 10:** Antimicrobial activity against fungi (*Aspergillus flavus*, *Penicillium verrucosum*).

Material	Fungi			
	*A. flavus*		*P. verrucosum*	
	N	R	N	R
Control foil	5.36 ± 0.22	/	5.19 ± 0.21	/
PE-2%CS + CSNPs THY	5.23 ± 0.16	0.13	4.20 ± 0.12 *	0.99
PP-2%CS + CSNPs THY	5.03 ± 0.18	0.33	3.83 ± 0.28 *	1.36
PE-2%CS + THYME	4.69 ± 0.14 *	0.67	3.12 ± 0.47 *	2.07
PP-2%CS + THYME	4.08 ± 0.22 *	1.28	3.08 ± 0.23 *	2.11
PE-2%CS + CSNPs ROS	1.34 ± 0.18 *	4.02	2.05 ± 0.24 *	3.14
PP-2%CS + CSNPs ROS	1.82 ± 0.62 *	3.54	2.32 ± 0.10 *	2.87
PE-2%CS + CSNPs CIN	1.09 ± 0.25 *	4.27	2.12 ± 0.24 *	3.07
PP-2%CS + CSNPs CIN	1.56 ± 0.33 *	3.80	3.00 ± 0.15 *	2.19

N—number of bacteria/spores per cm^2^ of material; R—antimicrobial activity. * *p* < 0.001 for Dunnett *t*-test.

**Table 11:** Sample description.

Sample Notation	Description of Sample
PE PP PE-UV/OZONE PP-UV/OZONE CS THY ROS CIN CSNPs CSNPs THY CSNPs ROS CSNPs CIN PE-2%CS + CSNPs THY PP-2%CS + CSNPs THY PE-THYME PP-THYME PE-2%CS + CSNPs ROS PP-2%CS + CSNPs ROS PE-2%CS + CSNPs CIN PP-2%CS + CSNPs CIN PE-2%CS PP-2%CS	Polyethylene foil Polypropylene foil PE foil treated with UV/ozone system PP foil treated with UV/ozone system Chitosan powder Thyme extract, *Thymus vulgaris* L. Rosemary extract, *Rosmarinus officinalis* L. Cinnamon extract Chitosan nanoparticle dispersion Dispersion of chitosan nanoparticles with encapsulated thyme extract Dispersion of chitosan nanoparticles with encapsulated rosemary extract Dispersion of chitosan nanoparticles with encapsulated cinnamon extract UV/ozone-treated PE foil, coated with 2% CS and CSNPs THY (2 layers) UV/ozone-treated PP foil, coated with 2% CS and CSNPs THY (2 layers) UV/ozone-treated PE foil, coated with thyme extract solution UV/ozone-treated PP foil, coated with thyme extract solution UV/ozone-treated PE foil, coated with 2% CS and CSNPs ROS (2 layers) UV/ozone-treated PP foil, coated with 2% CS and CSNPs ROS (2 layers) UV/ozone-treated PE foil, coated with 2% CS and CSNPs CIN (2 layers) UV/ozone-treated PP foil, coated with 2% CS and CSNPs CIN (2 layers) PE foil treated with UV/ozone, with 2% CS PP foil treated with UV/ozone, with 2% CS

## References

[1] Bastarrachea L.J., Wong D.E., Roman M.J., Lin Z., Goddard J.M. (2015). Active packaging coatings. Coatings.

[2] Yildirim S., Röcker B., Pettersen M.K., Nilsen-Nygaard J., Ayhan Z., Rutkaite R., Radusin T., Suminska P., Marcos B., Coma V. (2018). Active packaging applications for food. Compr. Rev. Food Sci. Food Saf..

[3] Drake P.L., Hazelwood K.J. (2005). Exposure-related health effects of silver and silver compounds: A review. Ann. Occup. Hyg..

[4] Torres-Arreola W., Soto-Valdez H., Peralta E., Cárdenas-López J.L., Ezquerra-Brauer J.M. (2007). Effect of a low-density polyethylene film containing butylated hydroxytoluene on lipid oxidation and protein quality of Sierra fish (Scomberomorus sierra) muscle during frozen storage. J. Agric. food Chem..

[5] Shahidi F., Zhong Y. (2010). Novel antioxidants in food quality preservation and health promotion. Eur. J. Lipid Sci. Technol..

[6] Aziz M., Karboune S. (2018). Natural antimicrobial/antioxidant agents in meat and poultry products as well as fruits and vegetables: A review. Crit. Rev. Food Sci. Nutr..

[7] Barbosa-Pereira L., Aurrekoetxea G.P., Angulo I., Paseiro-Losada P., Cruz J.M. (2014). Development of new active packaging films coated with natural phenolic compounds to improve the oxidative stability of beef. Meat Sci..

[8] Pokorný J. (2007). Are natural antioxidants better–and safer–than synthetic antioxidants?. Eur. J. Lipid Sci. Technol..

[9] Realini C.E., Marcos B. (2014). Active and intelligent packaging systems for a modern society. Meat Sci..

[10] Redondo-Cuevas L., Castellano G., Raikos V. (2017). Natural antioxidants from herbs and spices improve the oxidative stability and frying performance of vegetable oils. Int. J. Food Sci. Technol..

[11] Shahidi F. (2000). Antioxidants in food and food antioxidants. Food/nahrung.

[12] Donsì F., Annunziata M., Sessa M., Ferrari G. (2011). Nanoencapsulation of essential oils to enhance their antimicrobial activity in foods. LWT-Food Sci. Technol..

[13] Noshirvani N., Ghanbarzadeh B., Mokarram R.R., Hashemi M. (2017). Novel active packaging based on carboxymethyl cellulose-chitosan-ZnO NPs nanocomposite for increasing the shelf life of bread. Food Packag. Shelf Life.

[14] Özogul F., Öztekin R., Kulawik P. (2017). Biogenic amine formation and microbiological quality of anchovy (Engraulis encrasicolus) treated with lavender and lemon balm ethanol extracts. J. Food Sci..

[15] Pola C.C., Medeiros E.A., Pereira O.L., Souza V.G., Otoni C.G., Camilloto G.P., Soares N.F. (2016). Cellulose acetate active films incorporated with oregano (Origanum vulgare) essential oil and organophilic montmorillonite clay control the growth of phytopathogenic fungi. Food Packag. Shelf Life.

[16] Jayasena D.D., Jo C. (2013). Essential oils as potential antimicrobial agents in meat and meat products: A review. Trends Food Sci. Technol..

[17] Hyldgaard M., Mygind T., Meyer R.L. (2012). Essential oils in food preservation: Mode of action, synergies, and interactions with food matrix components. Front. Microbiol..

[18] Malhotra B., Keshwani A., Kharkwal H. (2015). Antimicrobial food packaging: Potential and pitfalls. Front. Microbiol..

[19] De Dicastillo C.L., Bustos F., Guarda A., Galotto M.J. (2016). Cross-linked methyl cellulose films with murta fruit extract for antioxidant and antimicrobial active food packaging. Food Hydrocoll..

[20] Glaser T.K., Plohl O., Vesel A., Ajdnik U., Ulrih N.P., Hrnčič M.K., Bren U., Fras Zemljič L. (2019). Functionalization of polyethylene (PE) and polypropylene (PP) material using chitosan nanoparticles with incorporated resveratrol as potential active packaging. Materials.

[21] Gutiérrez M.Q., Echeverría I., Ihl M., Bifani V., Mauri A.N. (2012). Carboxymethylcellulose–montmorillonite nanocomposite films activated with murta (Ugni molinae Turcz) leaves extract. Carbohydr. Polym..

[22] Stoleru E., Munteanu S.B., Dumitriu R.P., Coroaba A., Drobotă M., Zemljic L.F., Pricope G.M., Vasile C. (2016). Polyethylene materials with multifunctional surface properties by electrospraying chitosan/vitamin E formulation destined to biomedical and food packaging applications. Iran. Polym. J..

[23] Zemljič L.F., Plohl O., Vesel A., Luxbacher T., Potrč S. (2020). Physicochemical Characterization of Packaging Foils Coated by Chitosan and Polyphenols Colloidal Formulations. Int. J. Mol. Sci..

[24] Zemljič L.F., Tkavc T., Vesel A., Šauperl O. (2013). Chitosan coatings onto polyethylene terephthalate for the development of potential active packaging material. Appl. Surf. Sci..

[25] Han J.W., Ruiz-Garcia L., Qian J.P., Yang X.T. (2018). Food packaging: A comprehensive review and future trends. Compr. Rev. Food Sci. Food Saf..

[26] Milinčić D.D., Popović D.A., Lević S.M., Kostić A.Ž., Tešić Ž.L., Nedović V.A., Pešić M.B. (2019). Application of Polyphenol-Loaded Nanoparticles in Food Industry. Nanomaterials.

[27] Yang W., Owczarek J., Fortunati E., Kozanecki M., Mazzaglia A., Balestra G., Kenny J., Torre L., Puglia D. (2016). Antioxidant and antibacterial lignin nanoparticles in polyvinyl alcohol/chitosan films for active packaging. Ind. Crops Prod..

[28] European Commission Union Guidelines on Regulation (EU) No 10/2011 on Plastic Materials and Articles Intended to Come into Contact with Food. https://ec.europa.eu/food/safety/chemical_safety/food_contact_materials/legislation_en.

[29] Liu Q., Meng X., Li Y., Zhao C.-N., Tang G.-Y., Li H.-B. (2017). Antibacterial and antifungal activities of spices. Int. J. Mol. Sci..

[30] Fei L.U., Ding Y.C., Ye X.Q., Ding Y.T. (2011). Antibacterial effect of cinnamon oil combined with thyme or clove oil. Agric. Sci. China.

[31] Guynot M., Marin S., Setu L., Sanchis V., Ramos A. (2005). Screening for antifungal activity of some essential oils against common spoilage fungi of bakery products. Food Sci. Technol. Int..

[32] Riaz A., Lei S., Akhtar H.M.S., Wan P., Chen D., Jabbar S., Abid M., Hashim M.M., Zeng X. (2018). Preparation and characterization of chitosan-based antimicrobial active food packaging film incorporated with apple peel polyphenols. Int. J. Biol. Macromol..

[33] Nieto G., Ros G., Castillo J. (2018). Antioxidant and antimicrobial properties of rosemary (Rosmarinus officinalis, L.): A Review. Medicines.

[34] Abreu A.C., McBain A.J., Simoes M. (2012). Plants as sources of new antimicrobials and resistance-modifying agents. Nat. Prod. Rep..

[35] Calo J.R., Crandall P.G., O’Bryan C.A., Ricke S.C. (2015). Essential oils as antimicrobials in food systems–A review. Food Control.

[36] Ristić T., Hribernik S., Fras-Zemljič L. (2015). Electrokinetic properties of fibres functionalised by chitosan and chitosan nanoparticles. Cellulose.

[37] Ristić T. (2014). Antimicrobial Medical Textiles Based on Chitosan Nanoparticles for Gynaecological Treatment. Ph.D. Thesis.

[38] Helander I., Nurmiaho-Lassila E.-L., Ahvenainen R., Rhoades J., Roller S. (2001). Chitosan disrupts the barrier properties of the outer membrane of Gram-negative bacteria. Int. J. Food Microbiol..

[39] Bordes C., Bouarab-Chibane L., Forquet V., Lantéri P., Clément Y., Leonard L., Oulahal N., Degraeve P. (2019). Antibacterial properties of polyphenols: Characterization and QSAR (Quantitative structure–activity relationship) models. Front. Microbiol..

[40] Zhang X., Wang L., Levänen E. (2013). Superhydrophobic surfaces for the reduction of bacterial adhesion. Rsc Adv..

[41] Cuero R.G. (1999). Antimicrobial action of exogenous chitosan. Exs.

[42] Hernandez-Lauzardo A.N., Bautista-Baños S., Velazquez-Del Valle M.G., Méndez-Montealvo M., Sánchez-Rivera M., Bello-Perez L.A. (2008). Antifungal effects of chitosan with different molecular weights on in vitro development of Rhizopus stolonifer (Ehrenb.: Fr.) Vuill. Carbohydr. Polym..

[43] Rice-Evans C.A., Miller N.J., Paganga G. (1996). Structure-antioxidant activity relationships of flavonoids and phenolic acids. Free Radic. Biol. Med..

[44] López-Mata M.A., Ruiz-Cruz S., Silva-Beltrán N.P., Ornelas-Paz J.D.J., Ocaño-Higuera V.M., Rodríguez-Félix F., Cira-Chávez L.A., Del-Toro-Sánchez C., Shirai K. (2015). Physicochemical and antioxidant properties of chitosan films incorporated with cinnamon oil. Int. J. Polym. Sci..

[45] Klančnik A., Piskernik S., Jeršek B., Možina S.S. (2010). Evaluation of diffusion and dilution methods to determine the antibacterial activity of plant extracts. J. Microbiol. Methods.

